# Decision-making style explains the withdrawal behavior of shy individuals: evidence from Chinese college students

**DOI:** 10.3389/fpsyg.2023.1292096

**Published:** 2023-12-22

**Authors:** Yang Yu, Hong Sun

**Affiliations:** College of Teacher Education, Taishan University, Tai’an, China

**Keywords:** shyness, IGT, decision-making, withdrawal behavior, rewards and penalties

## Abstract

Few studies have examined the mechanisms linking motivated behavior and reward–punishment stimuli in shy individuals. This study was designed to probe these mechanisms by examining shy and non-shy college student responses to both monetary rewards and penalties in the Iowa Gambling Task (IGT). Specifically, out of the 280 undergraduates surveyed in East China, 45 participants (18 boys) identified as shy and 45 (19 boys) identified as non-shy based on their shyness questionnaire scores were selected to participate in the IGT. Results revealed that shy participants selected favorable low-risk seeking decks (deck C) more frequently and adverse high-risk seeking decks (deck B) less frequently and were more inclined to change deck selection after incurring a net loss. Furthermore, the net score of shy students was higher than that of nonshy students. Results demonstrated that shy people were the winners of IGT games, indicating that they are more likely to exhibit risk-averse behaviors when making decisions. The results are discussed from the perspective of the decision-making style and practical implications of shy individuals.

## 1 Introduction

Shyness involves a person’s excessive worry or anxious self-preoccupation over the evaluations of, punishments from, or unknown threats from others. It is a subconstruct of social withdrawal, which refers to an individual isolating themselves from their peer group (Rubin et al., 2009). However, due to the conflict between approach and avoidance in shy individuals (high approach–avoidance conflict), shyness differs from other social withdrawal constructs such as behavioral inhibition (low approach and high avoidance motivation) or social silence (low approach–avoidance motivation) (Rubin et al., 2009; Hassan et al., 2021). In addition, shyness and social anxiety exhibit significant overlap in terms of physiological aspects (e.g., blushing), cognitive features (e.g., social fear), and behaviors (e.g., avoidance of social situations) (Brook and Willoughby, 2019). Nevertheless, research indicates that the majority of shy individuals (82%) do not manifest symptoms of social anxiety disorder. This suggests that shyness is not a clinical or pathological concept but rather a widely distributed personality variable in daily life (Heiser et al., 2003). Numerous correlational studies have reported that shyness is associated with a series of social–emotional and social adjustment problems (e.g., peer victimization, loneliness) (Zhang et al., 2021). Other studies have demonstrated that shy individuals may lack opportunities to develop cognitive and coping abilities from social activities (Yu et al., 2019b). This suggests that shyness represents a noteworthy personality trait, playing a substantial role in shaping social adaptation and competency development. In this study, the term “shyness” is employed to encompass a broad range of experiences related to discomfort in social situations and behavioral inhibition, aiming to explore how rewards and punishments influence their motivational behavior. Additionally, this research has the potential to provide valuable insights into the study of social anxiety phenomena within community samples.

Recently, many studies have explored the performance of shy individuals in social context from the perspective of social situational awareness. For instance, many correlational studies have indicated the mediating role of fear of negative evaluation in the relationship between shyness or social anxiety and social behavior, suggesting that shy or socially anxious individuals are more sensitive to social threats and exhibit less adaptive and friendly social behavior due to fear of negative evaluation (Hassan et al., 2021). In an electroencephalogram (EEG) study, researchers found that shy individuals exhibited a larger P3 amplitude in response to both self-related and friend-related comments, suggesting that excessive emotional involvement in interpersonal evaluations may be a neurocognitive deficit in shy individuals (Yu et al., 2019a). In addition, researchers have found that shy individuals display increased emotional reactivity when exposed to faces conveying various emotions. This increased responsiveness is thought to result from their heightened yet distorted capability to detect potential social threats (Beaton et al., 2010).

However, studies have not accounted for the fact that positive and negative cues are both present in most social situations and have mainly focused on one (particularly negative cues) over another. Therefore, it remains unclear whether positive or negative situational elicitors have a greater effect on the development and maintenance of shyness and how they affect motivated behavior.

### 1.1 Shyness and decision-making

The exploration of personality traits and emotional dysfunction has witnessed an increasing reliance on decision-making and gambling tasks that encompass both incentives and penalties (Lerner et al., 2015). For shyness, Addison and Schmidt (1999) measured behavior and heart rate during a gambling task in a sample of more and less shy undergraduate women. Results revealed that relative to their less shy counterparts, more shy individuals were significantly more likely to choose cards with small bets but high profit probability and exhibited a greater increase in heart rate during the task. These findings suggest that women who are extremely shy may exhibit a cautious decision-making involving a high degree of emotional stimulation. In another event-related potentials experiment, researchers found that whether adolescents won or lost, cues and feedback evoked more positive late/early N2 amplitudes and more positive frontal P2/late feedback-related negativity amplitudes in shy adolescents compared with their less shy counterparts. This result suggests that shy adolescents are hyperattentive to both winning/losing clues and feedback (Lackner et al., 2014).

These findings suggest that due to strong incentives, shy individuals may experience high emotional involvement and exhibit an exaggerated tendency to be risk-averse in decision-making tasks involving rewards and penalties. However, research on this topic is limited, and the effects of rewards and punishment on the motivated behavior of shy individuals thus remain unclear. The mechanisms linking decision-making and shyness must therefore be further investigated.

### 1.2 Shyness and the Iowa Gambling Task

**Figure 1:**
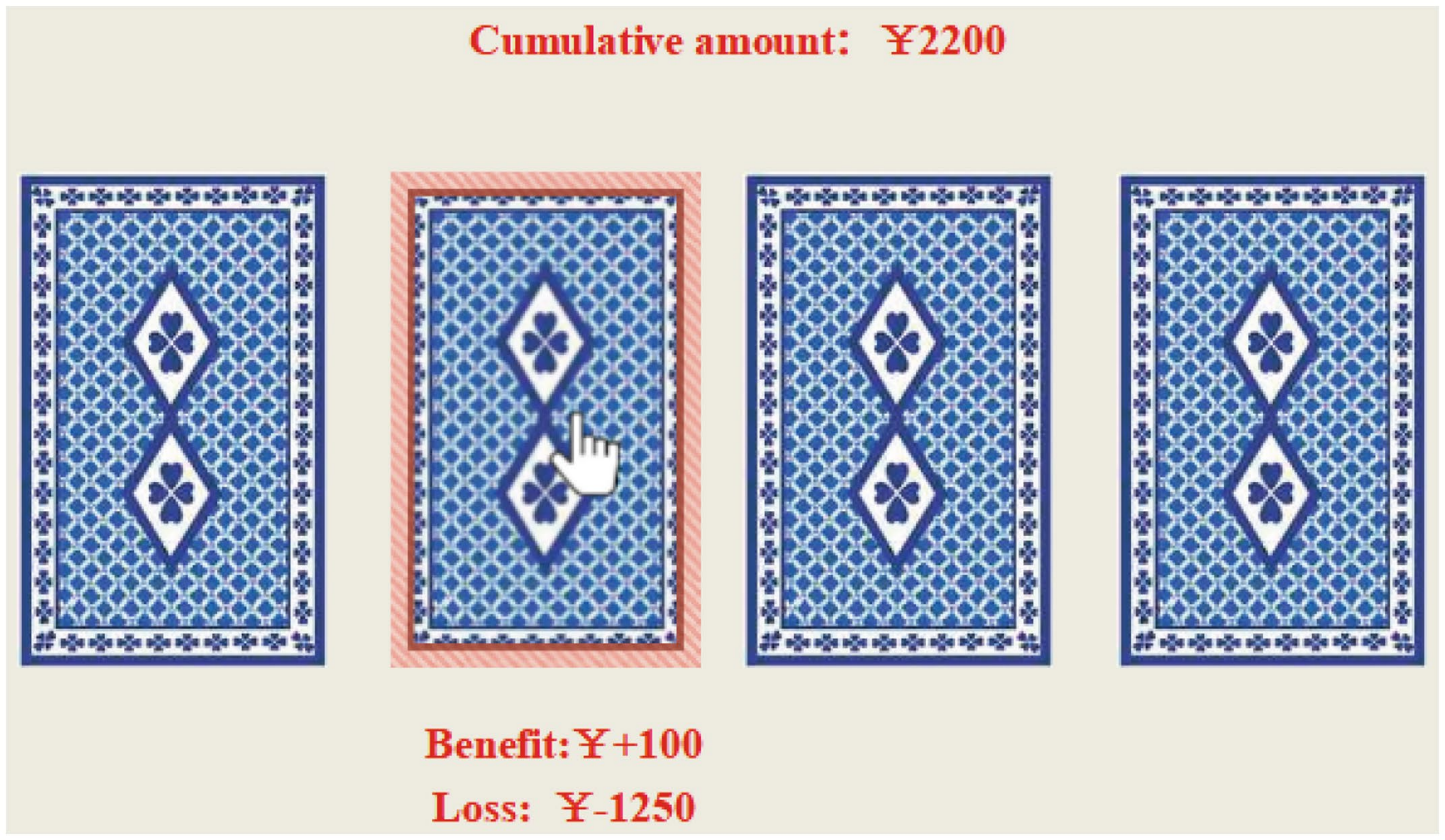
Program schematic of IGT.

Risky decision-making involves both emotion and logic and aims to make choices that are beneficial to individuals or others. The IGT is a traditional experimental task for measuring risky decision-making under ambiguity (Bechara et al., 1997). In the original version of the IGT, participants are initially given $2000 and told to maximize profit by selecting 100 cards from four decks. Participants enjoy high immediate gains ($100) once per selection from decks A and B. They incur five losses per 10 selections (high frequency, low intensity losses: $150, $200, $250, $300, $350) from deck A and one loss per 10 selections (low-frequency and high-intensity losses: always $1250) from deck B. Participants enjoy low immediate gains once per selection ($50) from deck C or D. They will incur five losses per 10 selections (high-frequency and low-intensity losses: always $50) from deck C and one loss per 10 selections (low-frequency and high-intensity losses: $250) from deck D. Therefore, decks A and B (bad decks) offer high immediate gains but long-term negative outcomes ($1,000 gains but $1,250 losses per 10 selections), and decks C and D (good decks) offer low immediate gains, but long-term positive outcomes ($500 gains but $250 losses on per 10 selections; Figure 1; Table 1). However, participants are not informed of the relative risks and benefits of each deck. They may become increasingly aware of these over time and guide remaining selections using the experience gained through feedback.

**Table 1:** Gain and loss structure of each deck.

Decks	Gains per choice	Losses per choice	Expectations for per 10 selection
A	100	0.5 probability loss 150, 200, 250, 300 or 350	−250
B	100	0.1 probability loss 1,250	−250
C	50	0.5 probability loss 50	+250
D	50	0.1 probability loss 250	+250

The IGT was originally used to measure decision-making in patients with damage to the ventromedial prefrontal cortices (VMPFC). In the IGT task, VMPFC-impaired patients continued to choose adverse high-risk seeking decks (decks A or B), whereas healthy individuals changed their preferences from adverse high-risk seeking decks to favorable low-risk seeking decks (decks C or D) after several selections (Bechara et al., 1997). Subsequent studies have found that high sensation seekers of various types or individuals from disinhibition groups (e.g., substance use disorder) are characterized by a tendency to make overly risky decisions. Individuals with this psychological characteristic tended to choose immediate profit and ignore losses (e.g., selecting decks A and B more). In other words, they may prioritize enjoying gains earlier and exhibit high risk tolerance. The IGT is thus useful in measuring cognitive impairment in high sensation seeking personality groups. However, few studies have examined whether it can demonstrate cognitive deficits in inhibited or low sensation seeking personality groups (e.g., shy individuals).

The IGT includes rewards and penalties and the likelihood of receiving either in each round is uncertain. Therefore, it induces ambiguity. This task may help researchers explore shy individuals’ decision-making function and sensitivity to rewards and punishment. In addition, researchers have proposed that rewards are related to approach motivation and positive emotions, whereas punishment is related to avoidance motivation and negative emotions (Lackner et al., 2014). Therefore, the IGT may help researchers understand the characteristics of shy individuals regarding self-awareness, emotional experience, and approach–avoidance conflict.

### 1.3 The present study

Research suggests that shy individuals exhibit hyperresponsivity to nonsocial monetary decision-making tasks (Addison and Schmidt, 1999; Lackner et al., 2014). However, relevant research is limited, and does not clearly demonstrate the effects of rewards and punishment on the motivated behavior of shy individuals. In this study, the classic IGT was used to explore the motivational behavior patterns of shy college students during risky decision-making. Because studies have found that shy individuals are motivated to avoid disapproval rather than gain approval in social scenarios (Hassan et al., 2021), we hypothesized that: (1) shy individuals exhibit more risk-averse behavior in the IGT, that is, more frequently choose favorable low-risk seeking decks (deck C and D) than adverse high-risk seeking decks (deck A and B). Additionally, in order to explore the practical implications of potential risk-averse behavior in shy individuals, we used net scores [the amount of (C + D)−(A + B)] as an indicator of risk-averse behavior and conducted a mediation analysis involving shyness, net scores, and social adaptation variables. Given that shy individuals frequently manifest a fear of punishment and avoidance behavior in real-life situations (Hassan et al., 2021; Xu et al., 2022), we hypothesized that: (2) relative to nonshy individuals, shy individuals are more inclined to change decks after net loss selections.

## 2 Methods

### 2.1 Participants

A total of 280 students from a university in East China participated in the survey. From this cohort, 45 individuals with high shyness scores (18 boys) and 45 with low shyness scores (19 boys) were identified to participate in the IGT game (see Procedures for participant sampling details). Age did not differ significantly between the two groups (*t*(1,88) = 0.64, *p* = 0.53, *cohen*’s *d =* 0.14). The shy group’s average shyness (*t*(1,88) = 21.30, *p* < 0.001, *cohen*’s *d = 4.49*) and general maladjustment (*t*(1,88) = 3.83, *p* < 0.001, *cohen*’s *d = 0.81*) scores were significantly higher than that of the nonshy group. All students were physically and mentally healthy, right-handed, and had normal or corrected-to-normal vision. The descriptive data of the sample is summarized in Table 2.

**Table 2:** Descriptive statistics of participants.

		Survey sample	Experimental sample	
			Shy group	Nonshy group
Homeplace	City	90	14	19
	Town	63	9	10
	Rural	117	22	16
Gender	Male	108	18	19
	Female	162	27	26
Age(*M* ± SD)		20.21 ± 0.79	20.34 ± 0.68	20.24 ± 0.74
Shyness(*M* ± SD)		37.73 ± 7.59	47.76 ± 4.08	27.53 ± 4.89
General maladjustment(*M* ± SD)		10.68 ± 5.02	12.71 ± 5.03	8.64 ± 5.01

### 2.2 Instruments

#### 2.2.1 Revised Cheek and Buss Shyness Scale

Cheek and Buss developed a scale to measure individuals’ feelings of discomfort and behavioral inhibition in the presence of others (Cheek, 1983). This scale has been revised and widely used among Chinese college students (Yu et al., 2019b). Example items include “I feel tense when I am with people I do not know well.” Participants were asked to respond on a 5-point Likert-type scale (1 = strongly disagree, 5 = strongly agree). The revised scale has 13 items and a higher score indicates a higher degree of shyness. In this study, the Cronbach’s α was 0.74.

#### 2.2.2 General maladjustment scale

The general maladjustment scale was developed by Welsh (1952). This scale measures a series of problems and emotional responses that indicate individuals’ adaptiveness to their environment (Mao and Dai, 2004). Example items include “I often feel pessimistic and disappointed.” The scale has 34 items that need to be answered yes or no (1 = yes, 0 = no) and a higher score represents a higher degree of maladjustment. This scale has been revised for and applied to individuals who are socialized in a Chinese cultural environment (Mao and Dai, 2004). In this study, the Cronbach’s α was 0.74.

#### 2.2.3 Iowa Gambling Task

The IGT involved four decks of cards (Section 1.2). Participants were told to choose cards from the four decks, and the purpose of the task was to win as much money as possible. To ensure that the four decks of cards were evenly distributed among the four chosen positions, 24 programs with different card sequences were created. Participants were then asked to randomly select one of these programs and participate in the subsequent game. After each selection, the benefits and losses of the selection and cumulative winnings were presented. The participants did not know that the task involved 100 trials distributed in five blocks. They could pause after completing each block until the end of the experiment. The participants’ choices were automatically recorded.

### 2.3 Procedures

The Ethics Committee granted approval for the administration of questionnaires and the experimental procedure involving human subjects. Initially, a series of questionnaires, encompassing demographic variables, shyness, and social maladjustment, was distributed to 280 Chinese undergraduates at a university in Shandong Province, East China. A total of 270 valid questionnaires were collected, achieving a commendable completion rate of 96.4%. Subsequently, the top 27% of students with the highest shyness scores were designated as the shy group, while the lowest 27% formed the nonshy group (Yu et al., 2019a). From these groups, 45 shy students (18 boys) and 45 nonshy students (19 boys) were randomly selected to participate in the experiment. All participants willingly volunteered for the experiment, receiving compensation in the range of 10 ~ 20 yuan. Prior to commencing, participants provided written informed consent. Following the survey, participants completed the Iowa Gambling Task (IGT) in a dimly lit and soundproofed room. The IGT comprised 5 blocks, and short breaks were allowed between each block to ensure participant comfort. The experiment utilized a cathode-ray tube display with specific viewing parameters, including a viewing distance of 80 cm, a horizontal viewing angle of 21°, and a vertical angle of 15°. Text in red font and images of four decks of cards were displayed on a gray screen (Figure 1). Upon completion of the experiment, participants were thanked and received a reward before leaving.

For this study, we employed a 2 × 4 × 5 mixed design with a between-participants factor (participant types: shy and nonshy) and two within-participants factors (4 decks, 5 blocks). The dependent variables for data analyses were the number of selections from each deck, net income/net loss selection conversion ratio, and net score. Net income referred to a greater amount of money won than lost in a single selection. Net loss referred to a greater amount of money lost than won in a single selection. The net income selection conversion ratio was the number of selections participants transferred to a different deck after receiving net income from one deck divided by the number of selections made receiving net income. The net loss selection conversion ratio was the number of selections participants transferred to a different deck after receiving net loss from one deck divided by the number of selections made receiving net loss. Net score was the number of selections from good decks C and D minus the number of selections from bad decks A and B.

## 3 Results

### 3.1 Comparison of number of selections from each deck

**Figure 2:**
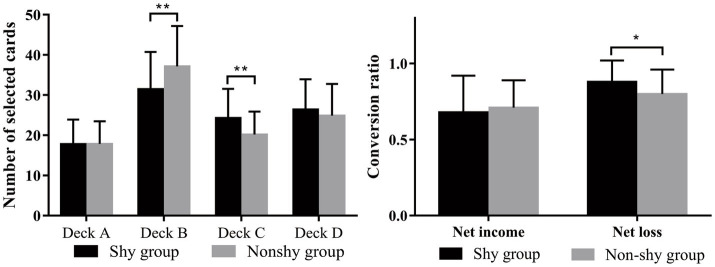
Comparison of shy and nonshy participant selected cards and conversion ratio.

The number of selections from each deck was analyzed using a 2 × 4 × 5 three-factor repeated-measures analysis of variance (participant types: shy and non-shy; 4 decks; 5 blocks). The main effect of participant type was not significant, *F*(1, 88) = 0.42, *p* = 0.52, *ηp2* = 0.005. The main effect of deck choice was significant, *F*(3, 264) = 57.21, *p* < 0.001, *ηp2* = 0.39. *Post hoc* analysis revealed that the selection numbers of the four decks were ordered as follows: A < C < D < B (*p* < 0.005). The interaction between participant types and decks was significant, *F*(3, 264) = 5.04, *p* = 0.004, *ηp2* = 0.05. A simple effects test was executed, and the results indicated that shy individuals selected deck B significantly less and deck C significantly more than nonshy individuals. Selections of deck A and D did not differ significantly between shy and nonshy individuals (Figure 2, left and Table 3). The main effect of blocks was not significant, *F*(4, 88) = 2.55, *p* = 0.08, *ηp*^2^ = 0.03. The interaction between participant types and blocks was not significant, *F*(4, 88) = 0.63, *p* = 0.53, *ηp*^2^ = 0.007. The interaction between decks and blocks was significant, *F*(12, 88) = 3.36, *p* = 0.001, *ηp*^2^ = 0.04, and detailed comparison results of the interaction are provided in the appendix. The interaction between participant types, decks and blocks was not significant, *F*(12, 88) = 0.31, *p* = 0.96, *ηp*^2^ = 0.004.

**Table 3:** Differences in deck selection results between shy and nonshy groups (M ± SD).

Decks	Shy group	Nonshy group	*p*
Deck A	17.80 ± 6.10	17.84 ± 5.64	0.971
Deck B	31.47 ± 9.27	37.13 ± 10.05	0.007
Deck C	24.31 ± 7.24	20.18 ± 5.73	0.003
Deck D	26.42 ± 7.51	24.91 ± 7.88	0.354

### 3.2 Net score results

Net scores were analyzed using a 2 × 5 two-factor repeated-measures ANOVA (two participant types: shy and nonshy; five blocks). The results indicated that the main effect of participant type was significant; the net score of the shy group was higher than that of the nonshy group, *F*(1,88) = 9.13, *p* = 0.003, *ηp2* = 0.09, *M*_shy group_ = 0.29 ± 5.93, *M*_non-shy group_ = −1.98 ± 6.07. The main effect of the five blocks was significant, *F*(4,88) = 6.54, *p* < 0.001, *ηp*^2^ = 0.07. *Post hoc* analysis demonstrated that the net scores for blocks 3, 4, and 5 were significantly greater than those of blocks 1 and 2 (*p* < 0.05). The interaction between participant type and blocks was not significant, *F*(4,88) = 0.22, *p* = 0.88, *ηp*^2^ = 0.002.

In studies using the IGT, net score has been the most common and effective index reflecting the decision-making function of participants (Bechara et al., 1997). Accordingly, this study examined the mediating effect of decision-making in the relationship between shyness (indicated by revised Cheek and Buss Shyness Scale score) and social maladjustment (indicated by general maladjustment scale score) by using net score as an index of decision-making function. Three variables were standardized, and the PROCESS macro in SPSS (v4) was used to examine the mediating effect. In results obtained using the bootstrap method (Hayes, 2013), the 95% bootstrap confidence intervals of the direct effect path (shyness→social maladjustment, *β* = 0.35^**^) and the indirect effect path (shyness→net score→social maladjustment, *β* = 0.07^**^) were [0.007, 0.17] and [0.15, 0.55], respectively. Neither interval included 0. These results indicate that shyness can positively predict social maladjustment through net score indicating risk-averse behavior.

### 3.3 Comparison of selection conversion ratios

Net income and net loss selection conversion ratios were analyzed using a 2 × 2 two-factor repeated-measures ANOVA (two participant types: shy and nonshy, two factors: net income and net loss). Results demonstrated that the main effect of participant type was not significant, *F*(1, 88) = 1.47, *p* = 0.23, *ηp2* = 0.02, *M*_shy group_ = 0.79 ± 0.19, *M*_non-shy group_ = 0.75 ± 0.17. The main effect of net income and net loss revealed that compared with obtaining net income, participants were significantly inclined to transfer to other decks after they chose one deck and obtained net loss, *F*(1,88) = 48.69, *p* < 0.001, *ηp2* = 0.37, *M*_net income_ = 0.70 ± 0.21, *M*_net loss_ = 0.84 ± 0.15. The interaction between these two independent variables was significant, *F*(1,88) = 4.66, *p* = 0.03, *ηp2* = 0.05. Simple effects test results demonstrated that net income selection conversion ratio did not significantly differ between shy and nonshy participants (*p* = 0.98, *M*_shy group_ = 0.70 ± 0.23, *M*_non-shy group_ = 0.70 ± 0.18, Cohen’s *d* = −0.13); however, after receiving a net loss, shy participants were significantly more inclined to transfer between decks (*p* = 0.01, *M*_shy group_ = 0.88 ± 0.14, *M*_non-shy group_ = 0.80 ± 0.16, Cohen’s *d* = 0.55) (Figure 2, right).

## 4 Discussion

This study examined differences in the IGT results of shy versus nonshy college students, and investigated the decision-making style in shy individuals. The results demonstrated that shy participants selected favorable low-risk seeking decks (deck C) more frequently, selected adverse high-risk seeking decks (deck B) less frequently, and the net score of the shy group was higher than that of the nonshy group. Furthermore, shy participants were more inclined to transfer between decks after net loss selections. These results supported the study hypotheses and indicated that shy individuals may be advantaged in the IGT. However, this selection pattern may reflect a unique pattern of cognition that underpins shyness and inhibited behavior.

### 4.1 Comparison of deck selection and net score results

Shy participants selected adverse high-risk seeking decks (deck B) less frequently and favorable low-risk seeking decks (deck C) more frequently than their nonshy counterparts. Correspondingly, the net score of the shy group was higher than that of the nonshy group. These findings support those in the literature (Addison and Schmidt, 1999; Lackner et al., 2014) and suggest that shy individuals exhibit greater risk avoidance in decision-making tasks where potential benefits and risks coexist. One possible interpretation of our findings is based on Asendorpf’s shyness model. Asendorpf proposes that shy people exhibit normal motivations to approach and socialize with others but are too fearful and withdrawn to actually do so (approach–avoidance conflict) (Hassan et al., 2021). Our results are consistent with this proposal. Specifically, in the IGT, shy individuals may initially desire to earn greater rewards, but the considerable penalties that accompany high rewards make the regulation of negative emotions difficult. Thus they eventually avoid these high-risk decks and choose low-risk decks. These results suggest that the approach–avoidance conflict that underpins shyness may not be specific to social situations and may generalize to less context-specific types of reward and punishment stimuli.

An alternative explanation could be linked to the behavioral tendencies of shy individuals to mitigate ambiguous emotional threats. Many theorists have proposed that emotions and personality serve an adaptive coordination purpose, triggering a set of responses (e.g., decision-making) that enable individuals to address encountered problems quickly. Accordingly, shy or anxious individuals experience discomfort or behavioral inhibition because of assumed ambiguous emotional threats, therefore, they may behave in a manner that reduces this type of uncertainty or risk (Lerner et al., 2015). In the IGT, compared with other decks, the large and infrequent penalties of deck B increase its ambiguity and risk, whereas the small and frequent penalties of deck C increase its certainty and acceptability. After selecting deck B and receiving large penalties, shy individuals may perceive a greater threat of failure; therefore, to reduce the emotional threat triggered by uncertainty, shy individuals may prefer to select deck B less frequently and deck C more frequently than their nonshy counterparts. Recent studies have found that individuals with pathological anxiety demonstrate clear avoidance biases in their decision-making, suggesting that this may be driven by a reduced propensity to take risks rather than a stronger aversion to losses (Charpentier et al., 2016). Our research supports this viewpoint and indicates that enhanced risk aversion is not limited to clinical samples of social anxiety but is also present in non-clinical social anxious groups.

The present findings indicate that in certain risk avoidance tasks, shy individuals tend to benefit more than nonshy individuals. This outcome provides insight into enhancing our understanding of the behavioral performance disparities among individuals with varying degrees of shyness. Numerous studies have demonstrated that shyness can lead to adverse adaptation outcomes, including heightened internalizing problems and peer victimization (Zhang et al., 2021). Nevertheless, other studies have also indicated that shyness may be associated with beneficial adaptation results in certain contexts, such as increased sociability and enhanced academic performance (Chen et al., 1995; Poole and Schmidt, 2019). From the perspective of evolutionary psychology, the “shy-bold continuum” is a basic behavioral characteristic that organisms (humans, animals) have developed in the struggle for survival (e.g., boldness) and for avoiding threats (e.g., shyness). Both traits also are key to the adaptive behavior in people’s social lives (Kagan et al., 1988). Accordingly, shy individuals may benefit from activities where risk avoidance is necessary, whereas nonshy individuals may benefit from activities requiring boldness in social situations. Because the IGT is used to investigate risky decision-making and is a task in which individuals who avoid risks benefit, our results indicate that shy individuals benefit more than non-shy individuals in activities that require risk avoidance.

However, with contemporary economic development and globalization, increasing numbers of activities require individuals to express themselves to gain support from others, especially in the college which is regarded as a highly competitive environment with excessively high standards for social performance (Fan et al., 2015). For shy students, the net score indicating risk-averse behavior may be related to approach–avoidance conflict and excessive behavioral inhibition that may be regarded as “self-reliance” or unfriendliness. Their decision-making function may therefore be a key cause of the social maladjustment in this competitive environment. If so, this decision-making function may disadvantage shy individuals in contemporary social environments (e.g., college) that tend to emphasize competitivity and proactivity. To confirm this viewpoint, this study examined the mediating effect of net score in the relationship between shyness (indicated by revised Cheek and Buss Shyness Scale score) and social maladjustment (indicated by general maladjustment scale score). Results indicate that shyness can positively predict social maladjustment through net score indicating risk-averse behavior. This finding implies that shy individuals’ decision-making function may lead to less beneficial outcomes in settings that prioritize competitivity and proactivity. Nevertheless, in analysis of variance, shyness is a categorical variable. Therefore, in the path analysis, only the shyness scores of the experimental group and the control group were taken into account, making the results of path analysis unreliable. Further research should investigate the link between decision-making function and social adjustment in shy individuals.

### 4.2 Comparison of selection conversion ratios

Results revealed that participants were significantly more likely to transfer between decks after receiving a net loss rather than a net gain. Using a simple choice task, Kubanek et al. (2015) found that rewards led to repetition of the previous choice, whereas punishment led to avoidance of the previous choice. Our results support this research and suggest that rewards and punishment are fundamentally distinct factors in governing behavior in decision-making activities.

More importantly, our results demonstrated that after incurring a net loss, shy participants were significantly more inclined to transfer between decks. Because rewards and punishment are related to approach motivation and avoidance motivation respectively, these results suggest that shy and nonshy individuals differ in avoidance motivation rather than approach motivation. Greater avoidance motivation may be key factor governing the behavior of shy individuals. In this study, the greater avoidance motivation of shy individuals may have been caused by net loss, making it difficult for them to regulate negative emotions and triggering avoidance behavior; therefore, they may have avoided previous choices and selected another deck. Similar to the experiment of this study, in a monetary incentive delay task (MID), shy and nonshy college students were required to respond rapidly to obtain a reward or to prevent a loss. Results revealed that shy students exhibited a large response discrepancy between reward and punishment stimuli, resulting from longer reaction times to punishment stimuli than reward stimuli. This indicated that shy individuals may exhibit normal motivation to approach and interact with others and simultaneously restrain themselves from doing so due to fear of potential punishment (Hardin et al., 2006). Our results support those of other studies and indicate that greater avoidance motivation may be a critical factor underlying shyness.

### 4.3 Limitations and future directions

This study has some limitations. First, the study was initially designed to compare responses to a decision-making task between individuals categorized as shy and non-shy. Consequently, we deliberately selected participants who exhibited extreme levels of shyness, both high and low, for the decision-making tasks. Nevertheless, this approach presents a challenge for subsequent mediation analysis since it lacks the participation of individuals with moderate levels of shyness, making the reliability of the mediation analysis results less certain. Future research endeavors should consider employing a larger sample size and more robust methodologies to delve deeper into the relationship between decision-making functioning and adaptive characteristics in individuals with shyness. Second, while this study primarily focused on shyness as the main research variable, its findings also have relevance for investigating social anxiety, extroversion, and other socially withdrawn groups within a community sample. To our best knowledge, these concepts often share measurement overlaps. However, with the progress of neuroscience and theoretical models, the comprehension of shyness has shifted from its behavioral and emotional aspects to encompass its biological origins and inner motivations. Furthermore, finer distinctions have been made between similar concepts (Rubin et al., 2009; Hassan et al., 2021; Zhang et al., 2021). For instance, despite sharing a common trait of fearfulness and anxiety, behavioral inhibition involves inhibited reactions toward both social and non-social stimuli, whereas shyness only occurs in social contexts. Despite the similarity in definition, social anxiety disorder is much more severe and intense than shyness (Rubin et al., 2009; Zhang et al., 2021). Future research should endeavor to offer empirical evidence confirming the distinctiveness of these closely related constructs by incorporating a greater variety of decision-making games and alternative tasks. Finally, this study has demonstrated that shy individuals are more likely to be risk-averse in ambiguous situations with both rewards and punishments, a pattern that could be seen in real-life social contexts. However, it is not known if the reactions of shy people in social contexts are similar to their responses in decision-making tasks. Consequently, it is essential for future research to establish a connection between the performance of shy people when faced with rewards and punishments and their social behavior.

Despite its limitations, this research contributes to our understanding of the motivated behavior and reward–punishment responses of shy individuals. First, our results supported previous research indicating that shy individuals may exhibit enhanced risk aversion in decision-making, therefore benefit from situations that require risk-averse behaviors. Enhanced risk aversion may not only be manifested in individuals with clinical social anxiety disorder but also in samples exhibiting shyness and social anxiety within the community (Charpentier et al., 2016). Secondly, this study emphasized that vital role of approach–withdrawal conflict in maintaining shyness-related behavior. Future research should verify the findings of this study in both social and nonsocial situations.

## Data availability statement

The original contributions presented in the study are included in the article/Supplementary material, further inquiries can be directed to the corresponding author.

## Ethics statement

The studies involving humans were approved by Academic Ethics Committee of Taishan University. The studies were conducted in accordance with the local legislation and institutional requirements. The participants provided their written informed consent to participate in this study.

## Author contributions

YY: Writing – original draft. HS: Writing – review & editing.
