# Supplementary material for: Decision-making style explains the withdrawal behavior of shy individuals: evidence from Chinese college students
Source: Front Psychol. 2023 Dec 22;14:1292096. doi: 10.3389/fpsyg.2023.1292096 (PMC10770851; doi:10.3389/fpsyg.2023.1292096)
Supplement: Supplementary file 2 [file Data_Sheet_1.PDF]

Table 1. The comparison results of the interaction between 4 decks and 5 blocks in IGT

Blocks	Decks(I)	Decks(J)	Mean Difference(I-J)	*SE*	Significance level	Upper 95%CI	Lower 95%CI
1	A	B	−3.24	0.43	0.00	−4.40	−2.09
		C	−0.28	0.25	0.86	−0.96	0.40
		D	−0.24	0.28	0.94	−0.99	0.50
	B	A	3.24	0.43	0.00	2.09	4.40
		C	2.97	0.41	0.00	1.86	4.07
		D	3.00	0.43	0.00	1.84	4.16
	C	A	0.28	0.25	0.86	−0.40	0.96
		B	−2.97	0.41	0.00	−4.07	−1.86
		D	0.03	0.20	1.00	−0.52	0.58
	D	A	0.24	0.28	0.94	−0.50	0.99
		B	−3.00	0.43	0.00	−4.16	−1.84
		C	−0.03	0.20	1.00	−0.58	0.52
2	A	B	−3.11	0.44	0.00	−4.29	−1.94
		C	−0.04	0.26	1.00	−0.74	0.66
		D	−1.09	0.38	0.03	−2.11	−0.07
	B	A	3.11	0.44	0.00	1.94	4.29
		C	3.07	0.41	0.00	1.95	4.18
		D	2.02	0.46	0.00	0.78	3.27
	C	A	0.04	0.26	1.00	−0.66	0.74
		B	−3.07	0.41	0.00	−4.18	−1.95
		D	−1.04	0.32	0.01	−1.91	−0.18
	D	A	1.09	0.38	0.03	0.07	2.11
		B	−2.02	0.46	0.00	−3.27	−0.78
		C	1.04	0.32	0.01	0.18	1.91
3	A	B	−3.19	0.44	0.00	−4.36	−2.02
		C	−0.58	0.34	0.43	−1.48	0.33
		D	−2.02	0.41	0.00	−3.12	−0.93
	B	A	3.19	0.44	0.00	2.02	4.36
		C	2.61	0.47	0.00	1.34	3.88
		D	1.17	0.50	0.13	−0.18	2.51
	C	A	0.58	0.34	0.43	−0.33	1.48
		B	−2.61	0.47	0.00	−3.88	−1.34
		D	−1.44	0.41	0.01	−2.56	−0.33
	D	A	2.02	0.41	0.00	0.93	3.12
		B	−1.17	0.50	0.13	−2.51	0.18
		C	1.44	0.41	0.01	0.33	2.56
4	A	B	−3.04	0.43	0.00	−4.21	−1.88
		C	−1.68	0.39	0.00	−2.73	−0.62
		D	−2.12	0.39	0.00	−3.16	−1.09
	B	A	3.04	0.43	0.00	1.88	4.21
		C	1.37	0.53	0.06	−0.05	2.78
		D	0.92	0.53	0.40	−0.49	2.34
	C	A	1.68	0.39	0.00	0.62	2.73
		B	−1.37	0.53	0.06	−2.78	0.05
		D	−0.44	0.47	0.92	−1.70	0.81
	D	A	2.12	0.39	0.00	1.09	3.16
		B	−0.92	0.53	0.40	−2.34	0.49
		C	0.44	0.47	0.92	−0.81	1.70
5	A	B	−3.89	0.53	0.00	−5.32	−2.46
		C	−1.84	0.40	0.00	−2.91	−0.78
		D	−2.37	0.43	0.00	−3.52	−1.22
	B	A	3.89	0.53	0.00	2.46	5.32
		C	2.04	0.66	0.02	0.28	3.81
		D	1.52	0.65	0.12	−0.22	3.27
	C	A	1.84	0.40	0.00	0.78	2.91
		B	−2.04	0.66	0.02	−3.81	−0.28
		D	−0.52	0.52	0.90	−1.91	0.87
	D	A	2.37	0.43	0.00	1.22	3.52
		B	−1.52	0.65	0.12	−3.27	0.22
		C	0.52	0.52	0.90	−0.87	1.91

Note: The number of selections from each deck was analyzed using a 2 × 4 × 5 three-factor repeated-measures analysis of variance (participant types: shy and non-shy; 4 decks; 5 blocks). The interaction between decks and blocks was significant, $F(12, 88)=3.36$, $p=0.001$, $\eta p^2=0.04$, and detailed comparison results of the interaction between decks and blocks are provided in the Table 1.
